# Supplementary material for: Analyzing the impact of sustainable economic development from the policy text network: Based on the practice of China’s bay area policy
Source: PLoS One. 2023 Dec 29;18(12):e0296256. doi: 10.1371/journal.pone.0296256 (PMC10756538; doi:10.1371/journal.pone.0296256)
Supplement: S1 Appendix — (DOCX) [file pone.0296256.s001.docx]

**Appendix A: List of covered pilot regions**

Names of government departments in Qianwan New Area:

A. DMC, District Management Committee

B. DEDB, District Economic Development Bureau

C. DFB, District Finance Bureau

D. DMSA, District Market Supervision Administration

E. DCB, District Commerce Bureau

F. DQTSB, District Quality and Technical Supervision Bureau

G. DHRSSB, District Human Resources and Social Security Bureau

H. DBEIT, District Bureau of Economy and Information Technology

I. DNRPCB, District Natural Resources and Planning and Construction Bureau

J. DDRB, District Development and Reform Bureau

Names of government departments in the Guangdong-Hong Kong-Macao Bay Area:

A.PG, Provincial Government

B. PDRC, Provincial Development and Reform Commission

C. PEITC, Provincial Economic and Information Technology Commission

D. PDST, Provincial Department of Science and Technology

E. PCA, Provincial Communications Administration

F. PBQS, Provincial Bureau of Quality Supervision

G. PDSITC, Provincial Network Security and Information Technology Committee

H. PDPE, Provincial Department of Public Education

I. PDHRSS, Provincial Department of Human Resources and Social Security

J. PDNR, Provincial Department of Natural Resources

K. PDIIT, Provincial Department of Industry and Information Technology

L. PDF, Provincial Department of Finance

M. PDEE, Provincial Department of Ecological Environment

N. PDT, Provincial Department of Transportation

O. PDC, Provincial Department of Commerce

P. PMSB, Provincial Market Supervision Bureau

Q. PTB, Provincial Taxation Bureau

R. PIPO, Provincial Intellectual Property Office

S. PPCPD, Provincial Party Committee Propaganda Department
