# Supplementary material for: Analyzing the impact of sustainable economic development from the policy text network: Based on the practice of China’s bay area policy
Source: PLoS One. 2023 Dec 29;18(12):e0296256. doi: 10.1371/journal.pone.0296256 (PMC10756538; doi:10.1371/journal.pone.0296256)
Supplement: Attachment — Submitted filename: 2-Response to Reviewers(ZHOU).docx [file pone.0296256.s002.docx]

Response to Reviewers

1.If you would like to make changes to your financial disclosure, please include your updated statement in your cover letter. Guidelines for resubmitting your figure files are available below the reviewer comments at the end of this letter.
Data Availability, Funding, Conflicts of interests have been added to the cover letter as follows:

(1)Data Availability: The authors confirm that all data underlying the findings are fully available without restriction. All relevant data are within the paper.

(2)Funding: This study was supported by Zhejiang Province Soft Science Research Project 2022C35101 and County Industrial Digitization Research Base (the sixth round of Ningbo Philosophy and Social Science Research Base). The funders had no role in study design, data collection and analysis, decision to publish, or preparation of the manuscript.

(3)Conflicts of interests: The authors declare that there are no conflicts of interest regarding the publication of this paper.

2.Please ensure that your manuscript meets PLOS ONE's style requirements, including those for file naming. The PLOS ONE style templates can be found at 
https://journals.plos.org/plosone/s/file?id=wjVg/PLOSOne_formatting_sample_main_body.pdf and 
https://journals.plos.org/plosone/s/file?id=ba62/PLOSOne_formatting_sample_title_authors_affiliations.pdf

The revised paper has been modified according to the format requirements of the template and the format of the published paper in PLOS one.

3.Thank you for stating the following in the Acknowledgments Section of your manuscript: 
"This work received support from Zhejiang Province Soft Science Research Project (2022C35101)."We note that you have provided funding information that is not currently declared in your Funding Statement. However, funding information should not appear in the Acknowledgments section or other areas of your manuscript. We will only publish funding information present in the Funding Statement section of the online submission form. 
Please remove any funding-related text from the manuscript and let us know how you would like to update your Funding Statement. Currently, your Funding Statement reads as follows: 
"There are no financial conflicts of interest to disclose.This work received support from Zhejiang Province Soft Science Research Project (2022C35101).The funders had no role in study design, data collection and analysis, decision to publish, or preparation of the manuscript."Please include your amended statements within your cover letter; we will change the online submission form on your behalf.

We have removed any funding-related text from the manuscript. Funding Statement has been placed in the cover letter as follows. Funding: This word was supported by Zhejiang Province Soft Science Research Project 2022C35101 and County Industrial Digitization Research Base (the sixth round of Ningbo Philosophy and Social Science Research Base). The funders had no role in study design, data collection and analysis, decision to publish, or preparation of the manuscript.

4.In your Data Availability statement, you have not specified where the minimal data set underlying the results described in your manuscript can be found. PLOS defines a study's minimal data set as the underlying data used to reach the conclusions drawn in the manuscript and any additional data required to replicate the reported study findings in their entirety. All PLOS journals require that the minimal data set be made fully available. For more information about our data policy, please see http://journals.plos.org/plosone/s/data-availability.

"Upon re-submitting your revised manuscript, please upload your study’s minimal underlying data set as either Supporting Information files or to a stable, public repository and include the relevant URLs, DOIs, or accession numbers within your revised cover letter. For a list of acceptable repositories, please see http://journals.plos.org/plosone/s/data-availability#loc-recommended-repositories. Any potentially identifying patient information must be fully anonymized.

Important: If there are ethical or legal restrictions to sharing your data publicly, please explain these restrictions in detail. Please see our guidelines for more information on what we consider unacceptable restrictions to publicly sharing data: http://journals.plos.org/plosone/s/data-availability#loc-unacceptable-data-access-restrictions. Note that it is not acceptable for the authors to be the sole named individuals responsible for ensuring data access.

We will update your Data Availability statement to reflect the information you provide in your cover letter.

Data Availability has been placed in the cover letter as follows. Data Availability: The authors confirm that all data underlying the findings are fully available without restriction. All relevant data are within the paper.There are not ethical or legal restrictions to sharing your data publicly.

5. Reviewer #1: The topic is interesting, and the author has done a great job in realizing the subject. However, there are few areas on the paper that is still lagging and should be addressed properly:
   (1)The paper language needs to be improved and proof read. I would suggest to the authors ask an native English speaker, or a professional agency to take on this role.

We have asked a professional English teacher to revise the language of the whole paper and correct some grammatical errors and unclear expressions, as detailed in the revised draft.

(2)The process of building policy networks needs to be more detailed

Thanks to anonymous experts for this advice. The specific steps to build the high-quality development policy text network for the manufacturing industry in the Bay Area are as follows: Step first, policy text collection. Step two, data preprocessing. Step three, policy entity identification based on deep learning. Step four, the construction of policy network model. Step five, policy network index design. Step six, policy text correlation calculation. How to operate each step is explained in detail, and the specific content is shown as follows:

Step first, policy text collection. Collection of policy texts refers to the process of collecting policy texts from various sources, including official government websites, news media and existing data sets[50]. This study conducted an empirical study on the high-quality development policy documents of the manufacturing industry issued by the administrative departments of Qianwan New Area and Guangdong-Hong Kong- Macao Greater Bay Area. In the collection of relevant policy texts, "Bay Area" and "manufacturing industry" were first taken as keywords, and then a Pathon crawler program based on request database was compiled. Finally, we logged in to the official websites of the administrative departments of Qianwan New Area and Guangdong- Hong Kong-Macao Greater Bay Area, and used the crawler to crawl the information under the policy section, including policy title, release time, release agency, and full text of the policy.

Step two, data preprocessing. The collected policy text is preprocessed, such as using regular expressions to realize Chinese text clauses, deleting illegal characters, and manually marking policy entities, so as to facilitate the follow-up research.

Step three, policy entity identification based on deep learning. Natural language processing knowledge and named entity recognition technology are used to dig deeply into policy texts, and key knowledge entities, such as government agencies and mentioned policies, as well as co-occurrence and inclusion relationships among entities, are identified from policy texts through semantic annotation and entity extraction, so as to achieve the goal of extracting structured knowledge from unstructured policy texts. The time span of the collected policy texts is 20 From January 2009 to December 2021 , the government departments of the two major bay areas searched for manufacturing policies, and collected 22 major policy texts in the two major bay areas, as shown in Table 1.

Step four, the construction of policy network model. Policy network model is a research method to analyze the interaction between diversified policy subjects in the policy process[51].This study is based on the dependency multi-layer network proposed by Buldyrev S et al.[52], and draws on the dependency network under intergovernmental relations[53], which organically combines the departmental cooperation network and the keyword co-occurrence network. The multi-layer network consists of more than two single networks, breaking through the restriction of homogeneity of nodes and edgeways, and including the membership network between nodes of different nature, which is organically combined with the cooperation network of government departments and the co-keyword network of high-quality development policy of manufacturing industry. Based on this, the correlation values among policy texts are quantitatively calculated to analyze the participation of Bay Area government departments in the policy formulation process and the focus of policy development. The policy network model constructed by the research is shown in Figure 1.

Step five, policy network index design. This paper conducts complex social network analysis for the manufacturing policies of Qianwan New Area and Guangdong-Hong Kong-Macao Greater Bay Area. Common network analysis metrics in this method are selected, including centrality index, structural hole index, network density index, etc. The definition and calculation methods of each index are as follows.

Step six, policy text correlation calculation. The policy text is calculated based on the dependency multi-layer network under the established inter-governmental relationship. This study uses the social network analysis method to explore the characteristics of high-quality development policy networks of the manufacturing industry in Qianwan New Area and Guangdong-Hong Kong-Macao Greater Bay Area from the perspectives of cooperation among policy participating departments, co-occurrence of policy keywords, comparison of overall cooperation closeness, comparison of keyword network characteristics, and correlation between departments and keywords.

(3)The sample could have been chosen to be more typical, and it is recommended that additional samples such as the Tokyo Bay Area and the San Francisco Bay Area, which have more mature Bay Area economies

New York Bay Area, San Francisco Bay Area and Tokyo Bay Area are the three major bay areas in the world, and this paper focuses on regional and country studies. The significance of the research is not only to have an in-depth understanding of a country or several regions, but also to put forward theoretical viewpoints with universal significance. The bay area study of China also breeds the possibility of producing theories of general significance. The above content has been added to the introduction to help readers understand the value of this study. In addition, the anonymous reviewer has put forward a good suggestion. In the next step, we will conduct a cross-country comparative study of Bay Area policy texts to understand the difference, way and learn from bay area texts in different countries.

Reviewer #2: This paper is generally well-written and addresses an intriguing and significant topic in energy economics literature. Despite the comprehensiveness and profundity of the research in this work, I have some suggestions for the authors to consider.
Must be discussed these points in the introduction section:
• How can machine-learning algorithms evaluate the urgency and tone of the sustainable development policy texts from China's Bay Area?
• How closely do the policy text networks resemble or deviate from global sustainable development objectives?
• What are the main hubs and operators in China's Bay Area policy text network for sustainable economic growth, and how have they changed over time?
• How do regional variations within the Bay Area of China show up in the network of texts on sustainable economic policy?

• Please do note that in the introduction section, you need to motivate the research area and find the research gap (what this study tries to fill).

The anonymous reviewers gave us good suggestions to add the above suggestions to the summary, as follows:

Text analysis is a data mining technology, mining content includes word frequency statistics, semantic analysis, topic analysis, sentiment analysis, entity recognition, relationship analysis, time series analysis, text classification and text clustering, which is used to discover new and useful information from the original text. In recent years, with the rapid development of the Internet and the arrival of the era of big data, the number of texts is also increasing, and information is mined from a huge number of original texts. More and more scholars begin to focus on machine learning and natural language processing technology to strengthen the ability of text analysis. Major techniques include Naive Bayes(NB)[1], K-Nearest Neighbor(KNN)[2], Support Vector Machines(SVM)[3], econometric methods[4], Conditional Random Fields (CRF)[5], and so on. The quantitative text analysis method based on machine learning provides an empirical and scientific tool for information mining of the texts of Bay Area economic policies and sustainable development policies. For example, through the network analysis of Bay Area sustainable development policy texts, we can understand the network hub nodes of policy texts, the characteristics of network evolution, and the differences of network structures in different bay areas. In order to better understand the reasons behind the policy and the future development trend, and provide effective suggestions for the implementation of the policy, bring new thinking and application value for the realization of the sustainable development goal of the Bay Area economy. To this end, we call for more, deeper, and more valuable machine learning quantitative text analysis to be put on the agenda in the future.

In addition, the content arrangement of each part of this paper is added in the introduction to improve readability, as follows:

This paper proceeds as follows: Section 2 is a literature review on the effect of industrial policy network and high-quality development of manufacturing industry in the bay area. Section 3 introduces the methodology, and it includes the model specification, policy data sources and design of policy network indicators. Results and discussion can be seen in Section 4-6. Section 4 reports policy network analysis results of the Qianwan New Area of Ningbo and Guangdong-Hong Kong-Macao Bay Area. Comparative analysis of the characteristics of the policy network in the two bay areas will be conducted in Section 5. Section 6 performs evaluation of high quality development system of manufacturing industry in bay area. The related research conclusions and the corresponding policy implications are included in Section 7.

Currently, from my perspective research area is not written properly to motivate the reader, the research gap is vague and even references are not enough to justify the topic. follow and cite some recent research papers such as " Investigating the interaction effect of urbanization and natural resources on environmental sustainability in Pakistan". 2) "Toward achieving environmental sustainability target in Organization for Economic Cooperation and Development countries: The role of real income, research and development, and transport infrastructure". 3) Linking economic growth and ecological footprint through human capital and biocapacity. 4) . Role of institutions in correcting environmental pollution: An empirical investigation. 5) Combined nonlinear effects of urbanization and economic growth on CO2 emissions in Malaysia. An application of QARDL and KRLS. 6) How do green energy investment, economic policy uncertainty, and natural resources affect greenhouse gas emissions? A Markov-switching equilibrium approach

References to the literature recommended by the review experts are listed below.

Policy is one of the important factors that affect the development of regional economy, which promotes sustainable development of regional economy by exerting intermediary effects. Domestic and foreign scholars have conducted extensive research on regional industrial policies[13,14], resource use policies[15], energy investment policies[16-18], R&D investment policies[19,20], environmental regulation policies[21,22], and other aspects. These studies have attracted widespread attention from policy makers and provided important references for building a mechanism for sustainable regional economic development.

15.Danish, Hassan S (2022) Investigating the interaction effect of urbanization and natural resources on environmental sustainability in Pakistan. International Journal of Environmental Science and Technology 20(8): 8477-8484. doi:10.1007/S13762-022-04497-X.

17.Danish, Zhang J, Hassan S, Iqbal K (2020) Toward achieving environmental sustainability target in organization for economic cooperation and development countries: The role of real income, research and development, and transport infrastructure. Sustainable Development 28(1): 83-90. doi:10.1002/sd.1973.

18.Hassan S, Bushra B, Muhammad S, Zhu B (2022) How do green energy investment, economic policy uncertainty, and natural resources affect greenhouse gas emissions? A Markov-switching equilibrium approach. Environmental Impact Assessment Review 97: 106887. doi:10.1016/J.EIAR.2022.106887.

21.Hassan S, Danish (2020) Role of institutions in correcting environmental pollution: An empirical investigation. Sustainable Cities and Society 53:101901. doi:10.1016/j.scs.2019. 101901.

22.Ashar A, Muhammad S, Tauseef H, Irfan K, Hashim K (2022) Combined nonlinear effects of urbanization and economic growth on CO2 emissions in Malaysia. An application of QARDL and KRLS. Urban Climate 46: 101342. doi:10.1016/J.UCLIM.2022.101342.

These points should be discussed in the policy and conclusion
• What ethical ramifications may policy text network analysis employing AI have, particularly in terms of sustainable economic development?
• How does policy text network analysis relate to current qualitative techniques for examining policy impact?
• What are the constraints of utilizing policy text network analysis to assess the effectiveness of policies for sustainable economic development?

In the conclusions and policy implications, the above content recommended by the review experts is discussed as follows.

Relatively speaking, the qualitative analysis of sustainable development policy texts is more explanatory, inductive and exploratory, while the quantitative text analysis based on artificial intelligence is often more macro, deductive and objective. However, the analysis based on words will undoubtedly lead to the segmentation of certain fixed phrases or important collocation in statistics. In the interpretation, because of the inability to restore the context, the meaning of some words may be simplified or misunderstood. Through social network analysis, this paper reveals the policy framework for the sustainable development of the manufacturing industry in the Bay Area, and provides a more three-dimensional understanding of the formation mechanism of policy effects, which has strong explanatory depth and initiative advantages. However, there is still a lack of effective quantitative evaluation model for evaluating the effectiveness of sustainable economic development policies by using policy text network analysis.
